# Occlusion of the Vagina: Operation: Recovery

**Published:** 1853-12

**Authors:** 


From the American Journal of the Medical Sciences

*Occlusion of the Vagina: Operation: Recovery.*

Dr. Storer read the following cases, sent to him by Dr. Brainard, of Chicago.

July 7, 1852, I visited Mrs. ——, a young married woman, æt. 19 years, in the central part of Wisconsin, on account of an obstruction of the vagina.

On attempting to pass the finger into the vagina, it was arrested at its very orifice. Immediately behind the carunculæ myrtiformes there was a firm cicatrix which entirely shut up the passage, there being a transverse band from side to side, both above and below which there was a slight depression scarcely half an inch deep. On introducing a catheter into the bladder, and the finger into the rectum, the septum seemed thin, and not as thick as the natural septum of the bladder and vagina. The uterus was felt distended, filling up the cavity of the pelvis, and rising so as to be felt in front two inches above the pubis. She was constantly affected with severe expulsive pains.

This young woman, who was but 19 years of age, and presented appearances of not fully developed womanhood, was married on the 8th of October, 1851. About the 20th of November following, she was attacked with great pain, hemorrhage, profuse discharge of pus and mucus. There was constitutional disturbance of the severest kind, and her life was despaired of. There followed a discharge of shreds and sloughs, horribly offensive, which continued several weeks. This attack was taken for an abortion, but without good reason. It was simply a violent case of acute vaginitis, from too frequent sexual connection with the sexual organs imperfectly developed. She informed me, in answer to inquiries, that coition was very painful, and became more and more so.

Pains, indicating the return of the menstrual period, occurred February 1, 1852, and recurred twice at four weeks' interval, without discharge. From that time. the pains were continuous up to the time the operation was performed, the uterus gradually enlarging.

Operation, July 8, 1852. The patient being placed in a suitable position, an incision was made from before backwards through the band before described, extending the diameter of the vagina. The sides being separated by the finger, it was found that the mucous membrane of the rectum could be separated from that of the bladder with the point of the finger, without great violence. When a band of tissue resisted, it was divided with a blunt-pointed bistoury.

The separation was freely made to the uterus.

Here, however, instead of finding the os uteri, nothing but a smooth, round tumour presented itself. The finger was carried over its surface, and the surface denuded for a space at least two and a half inches in diameter. Finding no mouth, I determined to make a puncture at a point where a slight elevation was felt, and where it was presumed the os had been situated before it sloughed away. Accordingly, I made an incision in the form of two-thirds of a circle an inch in diameter, raising up a piece attached towards the anterior part. There was a copious discharge of the menstrual blood. The wall of the uterus was thick, and gave the sensation of fibres in cutting. Passing the finger within, a slight depression was found opposite the little tubercle noticed externally, seeming to confirm the idea that this was a vestige of the neck.

After treatment, a tent, or pessary, one and a half inches in diameter, made of pieces of sponge strung together upon a stick, and covered with oiled silk, was passed into the artificial vagina. The point was sufficiently small to press into the opening of the uterus, and the whole long enough to project externally. The case was intrusted to the care of Dr. Robert W. Earll, to whom, and to Dr. Axtell, I was indebted for assistance.

Not a bad symptom occurred; no swellings, and but little inflammation. August 25, she was able to walk from her room without difficulty.

Four months after the operation, November 8, she was well, doing the work of the house, and able to ride several miles. The cicatrization was perfect, at least that was Dr. Earll's opinion.—The menses have occurred regularly without pain, excepting the first, August 20, which was painful. She is unwilling to dispense with the pessary, as she experiences a sensation of falling of the womb without it, even when lying. Sexual connection is not painful, and there is no tendency to contraction.

*May* 30, 1853.—She continued in the same satisfactory condition.

*Occlusion of the Vagina and Uterus.*—I was called, May 25, 1853, to visit a patient with the above difficulties.

She was a young and healthy woman, who was delivered of her first child in January, 1853. The labour was difficult, lasting 48 hours, and was finished by cephalotomy.

About the first of April, 1853, Mrs. C. discovered that the vagina was closed.

On examination, it was found, indeed, that a perfect closure existed, commencing immediately behind the orifice of the canal, and extending back as far as the finger in the rectum could reach.

Being consulted at the time it was discovered, I advised no operation to be performed until several menstrual periods had passed, and the uterus and vagina were distended in some degree with the fluid.

Early in May, they wrote me that such was the case, which I found afterwards was probably not so.

*May* 25, 1853. I performed the operation in the same manner as in the case of the former patient.

The vagina was entirely obliterated and adherent, except a small piece in front of the os uteri.

The os and cervix were found of their natural form and position; but the os was closed. There was no distention with menstrual fluid. An incision was made across the mouth of the womb, and a catheter passed into it. A large tent, one and a half inches in diameter, was passed into the vagina, and the patient put under the influence of a full dose of morphia.

*July* 7. I have not yet received a report of the progress of the case from the physicians who had charge of it.

